# Expanded Hemodialysis ameliorates uremia-induced impairment of vasculoprotective KLF2 and concomitant proinflammatory priming of endothelial cells through an ERK/AP1/cFOS-dependent mechanism

**DOI:** 10.3389/fimmu.2023.1209464

**Published:** 2023-09-19

**Authors:** Hongfan Zhao, Dashan Wu, Michael Adu Gyamfi, Pinchao Wang, Christian Luecht, Anna Maria Pfefferkorn, Muhammad Imtiaz Ashraf, Julian Kamhieh-Milz, Janusz Witowski, Duska Dragun, Klemens Budde, Ralf Schindler, Daniel Zickler, Guido Moll, Rusan Catar

**Affiliations:** ^1^ Department of Nephrology and Internal Intensive Care Medicine, at Charité Universitätsmedizin Berlin, corporate member of Freie Universität Berlin, Humboldt-Universität zu Berlin, and Berlin Institute of Health (BIH), Berlin, Germany; ^2^ Department of Surgery, at Charité Universitätsmedizin Berlin, Berlin, Germany; ^3^ Institute of Transfusion Medicine, at Charité Universitätsmedizin Berlin, Berlin, Germany; ^4^ Department of Pathophysiology, Poznan University of Medical Sciences, Poznan, Poland; ^5^ BIH Center for Regenerative Therapies (BCRT) and Berlin-Brandenburg School for Regenerative Therapies (BSRT), at Charité Universitätsmedizin Berlin, Berlin, Germany

**Keywords:** chronic kidney disease (CKD), end-stage renal disease (ESRD), uremic toxins, cytokine signaling, systemic inflammation, cardiovascular disease (CVD), Krüppel-like factor 2 (KLF2), and expanded hemodialysis therapy (HDx)

## Abstract

**Aims:**

Expanded hemodialysis (HDx) therapy with improved molecular cut-off dialyzers exerts beneficial effects on lowering uremia-associated chronic systemic microinflammation, a driver of endothelial dysfunction and cardiovascular disease (CVD) in hemodialysis (HD) patients with end-stage renal disease (ESRD). However, studies on the underlying molecular mechanisms are still at an early stage. Here, we identify the (endothelial) transcription factor Krüppel-like factor 2 (KLF2) and its associated molecular signalling pathways as key targets and regulators of uremia-induced endothelial micro-inflammation in the HD/ESRD setting, which is crucial for vascular homeostasis and controlling detrimental vascular inflammation.

**Methods and results:**

First, we found that human microvascular endothelial cells (HMECs) and other typical endothelial and kidney model cell lines (e.g. HUVECs, HREC, and HEK) exposed to uremic serum from patients treated with two different hemodialysis regimens in the Permeability Enhancement to Reduce Chronic Inflammation II (PERCI-II) crossover clinical trial - comparing High-Flux (HF) and Medium Cut-Off (MCO) membranes - exhibited strongly reduced expression of vasculoprotective KLF2 with HF dialyzers, while dialysis with MCO dialyzers led to the maintenance and restoration of physiological KLF2 levels in HMECs. Mechanistic follow-up revealed that the strong downmodulation of KLF2 in HMECs exposed to uremic serum was mediated by a dominant engagement of detrimental ERK instead of beneficial AKT signalling, with subsequent AP1-/c-FOS binding in the KLF2 promoter region, followed by the detrimental triggering of pleiotropic inflammatory mediators, while the introduction of a KLF2 overexpression plasmid could restore physiological KLF2 levels and downmodulate the detrimental vascular inflammation in a mechanistic rescue approach.

**Conclusion:**

Uremia downmodulates vasculoprotective KLF2 in endothelium, leading to detrimental vascular inflammation, while MCO dialysis with the novel improved HDx therapy approach can maintain physiological levels of vasculoprotective KLF2.

## 1 Introduction

Chronic kidney disease (CKD) and end-stage renal disease (ESRD) are a major public health burden despite recently improved global awareness and better access to kidney care (1–4). In addition to kidney transplantation (KTx) (5), several renal replacement therapies (RRTs), such as peritoneal dialysis (PD) (6) and hemodialysis (HD) (7), offer well-established life-sustaining therapies for ESRD patients (4), which are nonetheless still associated with a considerable need for improvements considering their long-term morbidity and mortality outcomes (8–12).

Even under optimal care, both, CKD and in particular more advanced ESRD patients, suffer from multiple strong cardiovascular disease (CVD) risk factors, which are a leading cause for the underlying high morbidity and mortality (13). These complications can project into the manifestation of chronic microvascular injury, which in turn promotes the cardiovascular and post-inflammatory risks and detrimentally affects patient morbidity and mortality in the long run (14, 15). Thus, in order to optimize existing RRT treatments, intense research is currently ongoing to stepwise overcome any evident RRT-related chronic complications (8–12).

In the case of HD, where uremic toxins are removed *via* membrane-filter systems, uremic interaction with the endothelium and incomplete removal of uremic macromolecules promotes endothelial dysfunction and systemic microinflammation (15, 16). However, the novel expanded hemodialysis therapy (HDx) approach (9–11), which aims for a more efficient removal of middle-sized uremic toxins through use of novel improved middle cut-off (MCO) dialyzers (17), may offer better endothelial protection than the traditional high-flux (HF) dialyzers (7, 17, 18). Thus, more detailed mechanistic studies in this scientific context are of great interest.

As shown in the PERCI-II-MCO study (NCT02084381) (17), compared to HF-dialyzed patients the uremic serum from MCO-dialyzed patients exhibits reduces levels of typical pro-inflammatory mediators and less maladaptive angiogenesis in functional readouts (7, 17). In the current study, we have investigated in detail the role of Krüppel-like factor 2 (KLF2), an important zinc-finger transcription factor expressed in endothelial cells (ECs) (19, 20), considering its putative role in regulating and maintaining vascular homeostasis in the HDx setting.

KLF2 maintains vascular homeostasis and protection against endothelial dysfunction and atherosclerosis by multiple mechanisms (20), e.g. by blocking leukocyte adhesion to endothelium, by inhibiting endothelial inflammation, and by modulating thrombotic pathways and detrimental vascular calcification (20–24). Importantly, KLF2 exerts inhibitory effects on various pro-inflammatory stimuli, such as interleukin-1β (IL-1β), tumor-necrosis-factor-α (TNF-α), transforming-growth-factor-β (TGF-β), and thrombin, thus suggesting a pleiotropic anti-inflammatory and vasculo-protective effect (20, 23, 25). In turn, KLF2-suppression can promote endothelial inflammation and damage of glomerular endothelium (20, 26, 27).

On the transcriptional level, KLF2 has been shown earlier to suppress pulmonary fibrosis and inflammation, and to strongly impede TGF-β signals in the endothelium, through the inhibition of the activator protein 1 (AP-1) (28, 29). However, the potential role of KLF2 in uremia-induced endothelial inflammation, maladaptation, and dysfunction has not been studied in detail yet. Indeed, the modulation of vasculo-protective KLF2 may provide both, an interesting prognostic to either monitor the impact of inflammation and vascular health in advanced CKD/ESRD patients, but also an interesting therapeutic target to restore vascular health and integrity in uremic patients within vascular crisis.

In this study, we have investigated in detail the effect of uremia-induced regulation of KLF2 on the impact of pro-inflammatory mediators and endothelial function. First of all, we found that endothelial KLF2 expression is strongly downmodulated by uremia compared to healthy serum. Mechanistically, we have explored the underlying signaling mechanisms, and indicating that KLF2 signaling is mediated *via* regulation of AP-1. Eventually, we found that KLF2 expression differs in ECs exposed to uremic serum from patients dialyzed with conventional HF membranes compared to novel MCO dialyzers, thus associating with a reduced release of proinflammatory mediators with the novel improved HDx therapy.

## 2 Methods

### 2.1 Study patient description and serum samples

The uremic serum samples were obtained during the Permeability Enhancement to Reduce Chronic Inflammation-II clinical trial (PERCI-II-MCO; ClinicalTrials.gov NCT02084381; https://clinicaltrials.gov/ct2/show/NCT02084381) (17). The clinical study was conducted in accordance with the ethical principles of the Declaration of Helsinki and approved by the Ethics Committees of the Martin-Luther-University Halle-Wittenberg and the Charité Berlin, and written informed consent was given prior to inclusion of subjects into the study, as outlined previously (7, 17). The mean age of the patients in the PERCI-II study was as follows: MCO-first (58.1 +/- 16.5 years, n=23 patients) and HF-first (59.8 +/- 16.5 years, n=25). More detailed study patient parameters, serum sampling procedures, and its experimental use are described in greater detail in our prior publications (7, 17). Briefly, for mechanistic experiments, samples of 20 patients were pooled at equal volumes to obtain a uremic serum pool (USP) and non-uremic serum from 14 healthy donors (Age 36 ± 9.2 years; nine males, five females) was collected at our department, to generate a healthy serum pool (HSP) for comparison (30).

### 2.2 Endothelial cell culture and experimental readout

The disposable cell culture plastic materials were purchased from Becton Dickinson (Falcon; Franklin Lakes, USA) and basic chemicals from Sigma (St Luis, USA). The specific blocking reagents employed in the cell culture experiments were as follows: AP1-blocker (AP1B; SR-11302), AKT-blocker (AKTB; MK-2206), ERK-Blocker (ERKB; PD-184352), all obtained from Tocris Bioscience (Wiesbaden Germany) (6, 7, 25). The basic media and buffers (e.g. phosphate buffered saline; PBS) and penicillin/streptomycin were purchased from Biochrom-AG (Berlin, Germany) and the fetal calf serum (FCS) from Invitrogen (Darmstadt, Germany). The following specific cell types and respective specialized culture media were used for cell culture expansion and different experimental readouts.

1) Human microvascular endothelial cells (HMECs; catalogue no. CRL-3243; purchased from ATCC^®^, Manassas, VA, USA) were cultured in MCDB131 basic media for HMECs (Invitrogen and Gibco, Waltham, MA, USA) supplemented with 5% FCS, 10 mM L-glutamine, 10 ng/ml recombinant human EGF, 10nM hydrocortisone, and 1% penicillin/streptomycin (100 U/ml and 100 ug/ml, respectively) (31).

2) Human umbilical vein endothelial cells (HUVECs; Cat. No.: P10961, purchased from Innoprot; Bizkaia, Spain; https://innoprot.com) were cultured in Endothelial Growth Medium for HUVECs (Ca No: CCM027 from Biotechne/R&D Systems, www.rndsystems.com) supplemented with 5% Endothelial Growth Supplement (ECGS; containing human FGF-basic, LR3-IGF-1, VEGF165, EGF, heparin, hydrocortisone, l-ascorbic acid, and FCS, as described in detail by the supplier) and 1% penicillin/streptomycin (100 U/ml and 100 ug/ml, respectively).

3) Human reticular endothelial cells (HRECs; Cat. No.: P10880, from Innoprot) were cultured in Endothelial Cell Growth Medium HRECs (Ca No: P60104 from Innoprot), containing endothelial basic medium, supplemented with 5% FCS, 1% of ECGS (as provided by manufacturer), and 1% penicillin/streptomycin (100 U/ml and 100 ug/ml, respectively).

4) Human embryonic kidney cells (HEK293; Cat. No.: CRL-1573, from ATCC^®^) were cultured in Dulbecco’s Modified Eagle Medium (DMEM; Cat. No.: 30-2003 from ATCC^®^) supplemented with 10% FCS, 2mM GlutaMax (Gibco), 1mM sodium pyruvate, 10mM HEPES, and 100 U/ml penicillin/100 ug/ml streptomycin (31).

For experimental readout, the different ECs were seeded at standardized densities (20.000 cells per well) and cultured in basic medium with 0.5% FCS, supplemented with or without 5% (vol/vol) HSP, USP, or different types of individual uremic patient serum fractions from the MCO study, as described in detail in figure legends and reported earlier (7). The cell viability was assessed by quantifying mitochondrial activity with the water-soluble tetrazolium (WST-8) salt assay according to the manufacturer’s instructions (PromoCell) (30).

### 2.3 Gene expression analysis with qRT-PCR and Profiler PCR arrays

Gene expression was assessed with reverse transcription and quantitative real-time polymerase chain reaction (qRT-PCR), as outlined earlier (30, 32–37). Total RNA was extracted by using the PerfectPure RNA Cultured Cell Kit (5 Prime, Hamburg, Germany), its concentration and purity was estimated with a spectrophotometer (Nanodrop; Thermo Fisher Scientific), and the RNA reverse transcribed into cDNA with random hexamer primers, and qRT-PCRs performed on a 7500 Fast Block Real-Time PCR system (Applied Biosystems). Human primer sequences can be found in **Table 1**. The specificity of the qRT-PCR reaction was verified with melting curve analysis and the relative amount of transcript calculated with the cycle threshold method, using the Applied Biosystems 7500 System v.1.2.3 software and gene expression normalized relative to the endogenous reference gene β2-microglobulin (30). The Human Inflammatory Response and Autoimmunity RT2 Profiler PCR Array kit (Qiagen, Cat. No: PAHS-077ZA-6) was used to evaluate the expression of an exploratory panel of n=84 selected genes involved in the inflammatory response, according to the manufacturer’s instructions, as reported earlier (33–35).

**Table 1:** Sequences of primers used in quantitative-real-time-PCR analysis.

Gene	Sequence Sense Primer 5’➔3’	Sequence Antisense Primer 3’➔ 5’
**TNF-a**	gACAAgCCTgTAgCCCATgT	gAggTACAggCCCTCTgATg
**UBC**	ATTTgggTCgCggTTCTTg	TgCCTTgACATTCTCgATggT
**c-FOS (GM)**	AggAgAATCCgAAgggAAAg	CTTCTCCTTCAgCAggTTgg
**c-JUN (GM)**	CCCCAAgATCCTgAAACAgA	CCgTTgCTggACTggATTAT
**KLF2 mRNA**	CTgCCgTCCTTCTCCACTTT	CCCATggACAggATgAAgTC
**AP-1 Oligo** **for KLF2**	CAG CCC TCT CTG AGG CTG GAG CCA	TGG CTC CAG CCT CAG AGA GGG CTG
**KLF2_pcDNA3**	TAC CGA GCT CGG ATC CAT GGC GCT GAG TGA ACC CA	GAT ATC TGC AGA ATT CCT ACA TGT GCC GTT TCA TGT G
**KLF2_2109 + 93**	TGGCCTAACTGGCCGGTACCAGCCATTTGGGGTAAGGTACTAT	TCT TGA TAT CCT CGA GCG GGG AGA AAG GAC GCG G
**KLF2_1109 + 93**	TGGCCTAACTGGCCGGTACCTGCCGCAACTCCAACTTCTCC	Same sequence as KLF2_2109 + 93 above
**KLF2_509 + 93**	TGGCCTAACTGGCCGGTACCCATGAATCCTGGAGACTCCA	Same sequence as KLF2_2109 + 93 above
**KLF2_109 + 93**	TGGCCTAACTGGCCGGTACCTTAGGCTGCGCCCGGAGC	Same sequence as KLF2_2109 + 93 above

TNFa, tumor necrosis factor alpha; UBC, Ubiquitin C; c-FOS, early response element; AP-1, activator protein-1; and Oligo, oligonucleotide; KLF2, Krüppel-like factor 2; KLF2_2109-109, different KLF2 promoter 5’-deletion luciferase plasmids.

### 2.4 DNA constructs, transient transfection and luciferase assay

The DNA constructs of progressive KLF2 5’-putative promoter region deletion fragments (KLF2_2109, KLF2_1109, KLF2_509, and KLF2_109) were cloned into luciferase plasmid vector (pGL4.10, Promega, Madison, WI, USA), and the isolated plasmids were sent for sequencing (LC Genomics, Houston, TX, USA) to ensure the correct length of promoter segments. The HMECs were cultured in 12-well plates at a density that allowed them to reach 70-80% confluence after 24 hours. Transfections were performed using the TurboFect transfection reagent (Fermentas, Darmstadt, Germany) according to the manufacturer’s instructions. The cells were transfected with the constructed reporter plasmid (400ng/well) and co-transfected with the reference pRL-TK renilla plasmid (20ng/well), or the KLF2 overexpression gene, as described earlier (7). Luciferase activity was assessed with the dual-luciferase reporter assay system (Promega, Madison, WI, USA) according to the manufacturer’s protocol. Luciferase activity was measured using a microplate luminometer (Fluostar Optima, BMG Labtech, Ortenberg, Germany) and normalized to background levels of Renilla luciferase activity from co-transfected control vectors. The primers sequences are provided in **Table 2**. The human KLF2 gene promoter region -509 to -110 (**Figure S1**; GenBank AY738222.1) was analysed with the PROMO virtual laboratory program for the presence and location of potential transcription factor binding sites (http://gene-regulation.com/pub/programs/alibaba2/), accessed on 23 of March 2023 (25).

**Table 2:** Antibodies and reagents used for EMSA and western blot.

Antibody	Target Antigen	Host	Code	Company	Dilution
**KLF2**	KLF2 protein	Rabbit	SAB210832-100UL	Sigma	1:500
**c-FOS**	c-FOS Protein	Rabbit	#4384	CST	1:1000
**p-AKT**	p-AKT Protein	Rabbit	#9272	CST	1:1000
**p-ERK**	pERK Protein	Rabbit	#9102	CST	1:1000
**GAPDH**	GAPDH protein	Mouse	#5G4	Hytest	1:50000
**2^nd^ Antibody Anti-Mouse**	Mouse antigen	Donkey	#715035150	Dianova	1:5000
**2nd Antibody Anti-Rabbit**	Rabbit antigen	Donkey	#711035152	Dianova	1:10000

KLF2, Krüppel-like factor 2; c-FOS, early response element; AKT, protein kinase B; MAPK/ERK, map-kinase/extracellular signal-regulated kinase; and GAPDH, glyceraldehyde 3-phosphate dehydrogenase.

### 2.5 Nuclear extracts, electrophoretic mobility shift assay, and immunoassays

Nuclear extracts were prepared using the NE-PER Nuclear and Cytoplasmic Extraction Kit and oligonucleotide probes labelled with Biotin 3’ End DNA Labeling Kit (ThermoFisher). For the EMSA (37), the following probe was used (promoter region provided in parentheses): AP-1 - 5’-CAGGCTTCACTGAGCGTCCGCAG-3’ (-441 to -464) (**Table 2**). Each EMSA binding mixture (20 µl) contained 5 µg of nuclear extract, 20 fmol of labeled double-stranded probe, 1 µg of poly (deoxyinosinicdeoxycytidylic) acid, and 2 µl of 10 x reaction buffer, and was incubated at room temperature for 30 min. Protein–DNA complexes were analyzed by electrophoresis in 6% non-denaturing polyacrylamide gels and visualized using a LightShift Chemiluminescent EMSA Kit (ThermoFisher). The Pierce BCA Protein Assay Reagent (Perbio Science, Bonn, Germany) was used to determine the protein concentration for NE-PER and Western Blot analysis.

Cell extracts for KLF2, c-FOS, pAKT and PERK1/2 protein detection in Western blot analysis were prepared as described earlier (38). The cell lysates were electrophoresed on sodium dodecyl sulfate (SDS)-polyacrylamide gels and analyzed by Western blotting using primary antibodies directed against the target proteins KLF2, c-FOS, pAKT and pERK1/2 (Merck-Sigma-Aldrich, Taufenkirchen, Germany, and Cell Signaling Technology, Frankfurt, Germany, respectively) and GAPDH (Hytest, Turku, Finland), and respective secondary peroxidase-conjugated IgG (Dianova, Hamburg, Germany) (**Table 2**). The bands were visualized with an Enhanced Chemiluminescence Detection System (Thermo Scientific) and analzed Image J 1.43 software (National Institues of Health, Bethesda, MD, USA). The levels of IL-6, IL-8, and TNF-α in the supernatant of HMEC-conditioned media were measure by enzyme-linked immune assay (ELISA) using respective antibody pair buffer kits (ThermoFisher Scientific, Waltham, MA, USA) according to the manufacturer’s instructions.

### 2.6 Statistical analysis

Data are expressed as mean ± SEM. Non-parametric data are presented as medians. Statistical analysis and visualization was performed using GraphPad Prism (GraphPad^®^, San Diego, US) and R (version 3.5.1). Analyses of multiple variables were performed by one-way analysis of variance with Student–Newman–Keuls post- or Kruskal–Wallis with Müller–Dunn post-test. A P-value < 0.05 was considered statistically significant.

## 3 Results

### 3.1 Uremic serum reduces the transcription and expression of KLF2 in HMECs

The expression of KLF2 in ECs confers endothelial integrity by regulating anti-inflammatory and antithrombotic effects (21), while reduced transcriptional activity of KLF2 exacerbates chronic inflammation and endothelial damage in renal diseases (39). Thus, it is of great interest to study in detail the signaling events and functional effects related to KLF2.

**Figure 1:**
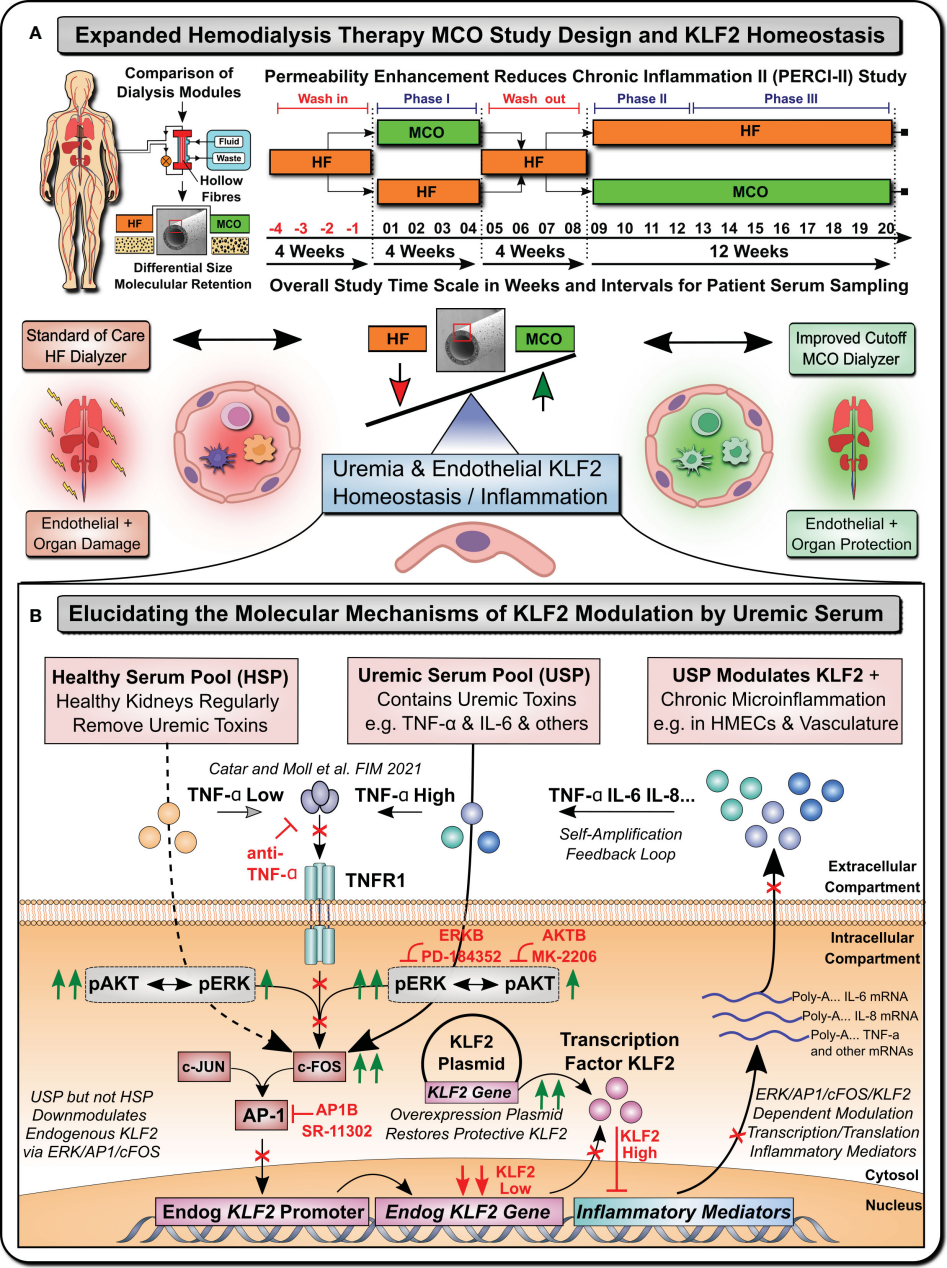
Uremia Modulates Vasculoprotective KLF2 Expression (Graphical Abstract). **(A)** Expanded Hemodialysis Therapy (HDx) MCO Study Design and KLF2 Homeostasis: Recently, particular attention has been placed into lowering chronic treatment-associated adverse cardiovascular diseases (CVD) and new optimized treatment concepts, such as “Expanded Hemodialysis Therapy” with improved molecular cut-off hemodialyzers (7, 9, 10). Within the PERCI-II study n=48 hemodialysis patients underwent crossover randomized multi-center comparison employing novel medium-cut-off (MCO; MCOI-Ci400, Gambro) dialyzers in comparison to standard of care high-flux (HF) hemodialyzers (PERCI-II-MCO; ClinicalTrials.gov: NCT02084381) (7, 17). These novel MCO dialyzers have an improved molecular size cut-off, which positively modulates systemic microinflammation and may thus be protective to lower uremia associated endothelial dysfunction and organ damage (7, 17). A key factor in regulating endothelial homeostasis and inflammation is Krüppel-like factor 2 (KLF2), which is the main focus of this mechanistic follow-up study to the PERCI-II clinical trial (7). **(B)** Elucidating molecular mechanisms of vasculoprotective KLF2 modulation by uremic serum: During the biomarker follow-up of the initial PERCI-II clinical trial KLF2 was identified as a potential target for mechanistic follow-up of the molecular signalling mechanisms underlying KLF2 impairment by uremia associated with the conventional HF dialysis regimen, but its improved KLF2 maintenance or restoration with the novel improved MCO dialyzer approach. For the mechanistic studies, to have sufficient amounts of standardized uremic serum available, we compared in detailed signalling pathway analysis and concomitant blocking experiments the response of microvascular endothelial cells (HMECs) to either healthy serum pools (HSP) or uremic serum pools (USP), as described in more detail in an earlier publication that identified TNF-signalling/triggering as a crucial mediator of USP activation in HMECs (7). In the current study, we first-of-all documented a strong impairment of KLF2 expression and concomitant pleiotropic upregulation of a panel of proinflammatory mediators in response to USP but not HSP, thereby triggering a vicious self-amplification loop to trigger the release of more inflammatory uremic toxins from HMECs (e.g. TNF-a, IL-6 etc). Signalling pathway analysis found that this was mediated by preferential engagement of map-kinase/extracellular signal-regulated kinase (MAPK/ERK) instead of protein kinase B (AKT) signalling by employing the respective molecular inhibitors (ERKB: PD-184352 and AKTB: MK-2206). Next, we found that this is mediated by modulation of activator protein 1 (AP-1) and early response element (c-FOS) transcription factor complex (AP-1/c-FOS) signalling (Employing AP1B: SR-11302) and its concomitant binding to the respective promoter region in endothelial KLF2 gene. Importantly, the introduction of a KLF2 overexpression plasmid could restore physiological KLF2 levels and downmodulate the detrimental vascular inflammation in a mechanistic rescue approach. These findings provide new avenues for molecular targets and treatment modalities to reduce chronic microinflammation in the context of hemodialysis.

For this purpose, we established a model system (**Figure 1**), to study the KLF2 response of microvascular HMECs to uremic serum pool (USP) from ESRD patients, as compared to healthy serum pool (HSP), at both the transcriptional mRNA and the translational protein level, employing USP from a well-defined clinical study cohort that has tested novel medium cut-off (MCO) dialyzers with increased molecular cut-off (**Figures 1A, B**) (7, 17, 18).

**Figure 2:**
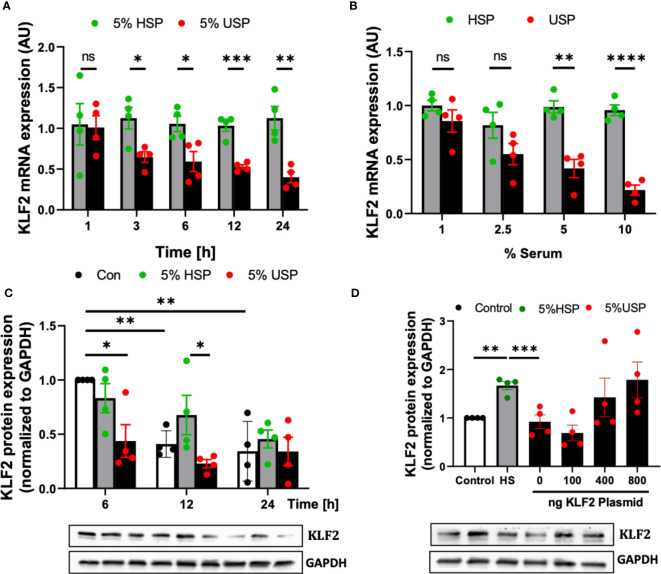
Uremic serum inhibits transcription and translation of KLF2 in HMECs. **(A)** Time-dependent downregulation of KLF2 upon USP exposure: KLF2 mRNA expression levels detected in HMECs (AU, arbitrary units, n=4) upon stimulation with either 5% healthy serum pool (HSP) or 5% uremic serum pool (USP), with strong downmodulation of KLF2 mRNA at 3-24 hours (50% reduction at 6 hours); **(B)** Dose-dependent downregulation of KLF2 upon HMEC exposure to USP: KLF2 mRNA expression levels (AU, n=4) after a 6-hours exposure of HMECs to USP or HSP at different serum doses (1%, 2.5%, 5% and 10%), respectively, verifying a strong downmodulation of KLF2 upon exposure to 2.5-10% USP; **(C)** Western blot quantification of time-dependent expression changes of KLF2 protein: KLF2 protein is shown relative to glyceraldehyde-3-phosphate dehydrogenase (GAPDH) house-keeping gene control (AU, n=4) upon cell exposure to either 5% HSP or 5% USP for either 6, 12, and 24 hours (optimal differential readout at 6 hours); and **(D)** Antagonizing KLF2 Downmodulation by USP: Western blot quantification of KLF2 protein expression in HMECs (AU, n=4) after transfection with different concentrations of KLF2 plasmids (100, 400 and 800 ng of KLF2 plasmid) to counteract the KLF2 downmodulation observed upon stimulation with 5% USP. Statistical comparison with ANOVA, Mean ± SEM, with *P<0.05, **P<0.01, ***P<0.001, and ****P<0.0001, ns, not significant.

First of all, we found a continuous time-dependent downmodulation of KLF2 mRNA in HMECs after 3-24 hour exposure to 5% USP, but not 5% HSP, with a 50% reduction as early as 6 hours post initiation (and 64% decrease by 24 hours), which was thus chosen as the default optimal exposure time for the assessment of changes in KLF2 mRNA transcription in the subsequent experiments (P<0.05 and P<0.1, respectively, **Figure 2A**).

Next, we tested HMEC exposure to different doses of serum and found that a 6-hour incubation with 5-10% USP, but not similar doses of HSP, led to a strong downmodulation of KLF2 mRNA transcript (around 50% reduction with 5% USP, P<0.01, and more than 80% reduction with 10% USP, P<0.001, **Figure 2B**). Due to the scarcity of these samples, we chose 5% USP as our standard working concentrations for the subsequent experiments.

Similarly, the exposure of HMECs to 5% USP resulted in a 50% and 66% reduction in KLF2 protein levels at 6 and 12 hours compared to HSP (Both P<0.5, **Figure 2C**). These results indicate that USP-exposure represses both, the transcriptional and post-transcriptional activity of KLF2 in endothelial cells, which may ultimately aggravate renal injury.

To better understand the mechanism of USP-induced inhibition of KLF2 activity, an exogenous pcDNA3.0-KLF2 (KLF2-OE) plasmid was constructed for this study and applied in our model to restore the inhibition of KLF2 induced by USP (**Figure 2D**). An amount of 400 ng of plasmid transfected into 50.000 HMECs was found to be the optimal amount to restore KLF2 protein levels in the USP compared to the HSP group.

### 3.2 Uremic serum induces KLF2 promoter inhibition via AP-1/c-FOS signaling

KLF2 transcriptional activity can be influenced by many regulatory elements and co-dependent genes, including the ubiquitous activator protein-1 (AP-1/c-FOS) family (40–42). Typical transcription factors and signaling cascades that may influence KLF2 transcription and consequent down-stream-signaling in HMECs may include cellular signaling pathways involving the transcription factor AP-1/c-FOS, but also the protein kinase B (AKT) and map-kinase/extracellular signal-regulated kinase 1/2 (MAPK/ERK1/2) signaling (7, 32).

**Figure 3:**
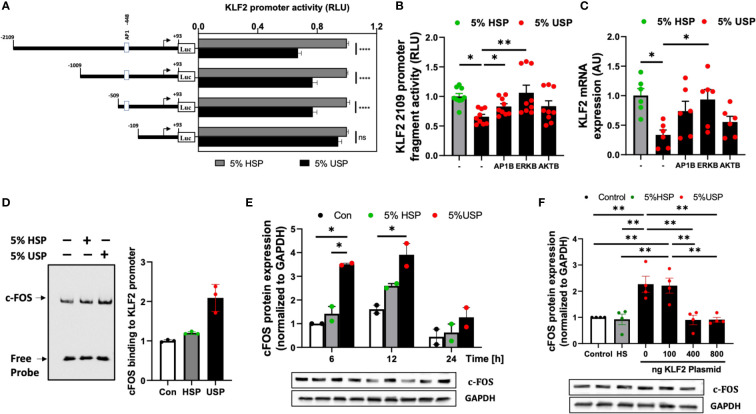
USP-inhibition of KLF2 in HMECs occurs in AP-1/c-FOS dependent manner. **(A)** KLF2 Promoter Analysis: Luciferase reporter assays indicate a functional loss of KLF2 activity upon deletion of the -509 to -109 promoter region containing the AP1-/c-FOS transcription factor binding site. HMECs were first transfected with different KLF2 reporter plasmid constructs containing progressive promoter region 5’-deletions, employing either the full-length -2109 construct, or different deletion constructs -1009, -509, -109, and +93, and then incubated with either 5% uremic serum pool (USP) or 5% healthy serum pool (HSP) for 6 hours, to quantify KLF2 promoter activity *via* readout of luciferase activity (RLU, relative luciferase activity, n=12 independent experiments); **(B, C)** Signaling Pathways: Inhibition of KLF2 expression upon a 6-hour exposure to 5% USP can be partially restored by blocking different signaling pathways: e.g. activator protein 1 (AP1B: SR-11302, 1000 nM), protein kinase B (AKTB: MK-2206, 100 nM), or map-kinase/extracellular signal-regulated kinase (MAPK/ERKB: PD-184352, 100 nM) with respective blocking agents (1-hour preincubation) (7, 25, 30), **(B)** Luciferase activity of HMECs containing full-length KLF2 reporter constructs (RLU, n=6) and **(C)** Expression of KLF2 mRNA (AU, arbitrary units, n=6); **(D)** AP-1/c-FOS transcription factor-binding site in the KLF2 promoter region: Biotin-labelled double-stranded oligonucleotides targeting the calculated AP-1/c-FOS positions -464 to -441 of the corresponding KLF2 promoter region were validated using the gel electrophoresis mobility shift assay (EMSA, one representative experiments is shown); **(E)** USP exposure induces a time-dependent upregulation of c-FOS expression in HMECs: Western blot analysis to quantify the expression of c-FOS protein in HMECs (AU, relative to GAPDH, n=2) which were left either untreated (baseline), or incubated for different time intervals (6, 12, and 24 hours) with either 5% USP or 5% HSP, demonstrating the strongest differential response at 6-12 hours; and **(F)** KLF2 overexpression downmodulates c-FOS expression upon USP exposure of HMECs: Different concentrations of KLF2 containing plasmid (100, 400 and 800 ng) were transfected into HMECs, followed by a 6-hour stimulation with 5% USP or 5% HSP, and western blot detection of KLF2 protein expression (AU, relative to GAPDH, n=4). Statistical comparison with ANOVA, Mean ± SEM, with *P<0.05, and **P<0.01, ns, not significant.

Employing our well-established luciferase-reporter-system (7), we found that treatment of HMECs containing full-length KLF2 promoter constructs with 5% USP, but not 5% HSP, resulted in a strong decrease of luciferase activity in the USP-group (P<0.001, **Figure 3A**), while stepwise 5’-deletions of the KLF2 promoter region demonstrate that the deletion of positions from -509 to -109 could fully restore the USP-stimulated inhibitory effect on KLF2 promoter activity (Line 1-3, P<0.001, vs. line 4, ns, **Figure 3A**). These results suggested that key regulatory elements for KLF2 transcription are located in the -509 to -109 region.

Next, we employed AP-1-, AKT-, and ERK-targeted pharmacological blockade to identify the signaling pathways in HMECs transfected with full-length KLF2-2109 promoter construct that are responsive to stimulation with 5% USP vs. 5% HSP baseline control. The blockade of AP-1 and ERK signaling most effectively restored the USP-induced downmodulation of KLF2 promoter activity (P<0.05 and P<0.01, **Figure 3B**) and KLF2 mRNA expression (P<0.05, **Figure 3C**), while blockade of AKT was less effective.

This demonstrates that the USP-induced decrease in KLF2 expression and activity that is depended on the promoter regions -509 to -109 bp engages both AP-1 and ERK signaling. Since AP-1 is a combinatorial transcription factor, the observed promoter region of KLF2 (Entailing the -509 to -109 bp promoter region) was analyzed using the PROMO virtual laboratory (http://alggen.lsi.upc.es/cgi-bin/promo_v3/promo/promoinit.cgi?dirDB=TF_8.3) to determine the DNA-binding switch between AP-1 transcription factor and KLF2 gene (40, 43), and we found that AP-1/c-FOS is a likely candidate could mediate AP-1 action on KLF2.

The specificity of the AP-1/c-FOS binding sites in the KLF2 promoter was confirmed by EMSA (**Figure 3D**). Semiquantitative EMSA analysis revealed that nuclear extracts from HMECs stimulated with 5% USP contained a DNA-protein complex with more efficient c-FOS probe binding compared to that in the HSP or unstimulated group (**Figure 3D**). We observed an approximately 2-fold increased intensity of c-FOS-probe in 5% USP-stimulated cells compared to 5% HSP stimulated cells and an approximately 3-fold increased intensity compared to unstimulated HMECs relative to the amount of free probe (P<0.05, **Figure 3D**). Computational analysis of probe sequences in the EMSA revealed that an AP-1/c-FOS DNA-protein binding site is located at the promoter region -464 to -441 bp (**Figure S1**).

Time-dependent stimulation of HMECs with 5% USP demonstrated a strong 2-3-fold up-regulation of c-FOS protein compared to stimulation with 5% HSP or untreated baseline, at 6 and 12 hours, but not at 24 hours (First two P<0.05, **Figure 3E**). Furthermore, we found that transfection and overexpression of 400-800 ng of KLF2 plasmid was effective to reverse the AP1-/c-FOS activity seen with 5% USP stimulation down to the level seen with 5% HSP or baseline control (P<0.01 for both 400 and 800 ng of KLF2 plasmid, **Figure 3F**).

Altogether, these data indicate that USP-induced inhibition of KLF2 includes binding of AP-1/c-FOS within the KLF2 promoter region -469 to -446 bp (**Figures 3A–D**), that the AP-1/c-FOS engagement is strongest at 6-12 hours post USP-stimulation (**Figure 3E**), and that it could be antagonized by overexpression of KLF2 plasmid in HMECs (**Figure 3F**).

### 3.3 Uremic serum modulates KLF2 via c-FOS, AKT and MAPK/ERK signaling

Another important component of AP-1 signaling is c-JUN, that can form the alternative transcription factor complex AP1-/c-JUN, as contrasted to the AP-1/c-FOS complex (25, 32, 44). In addition, it is known that ERK activation contributes to EC homeostasis and contributes to the release of inflammatory factors (7, 25, 45), while the role of AKT-signaling in HMECs, is not yet clearly elucidated in the uremic state and conflicting results were reported in the past (46–48).

**Figure 4:**
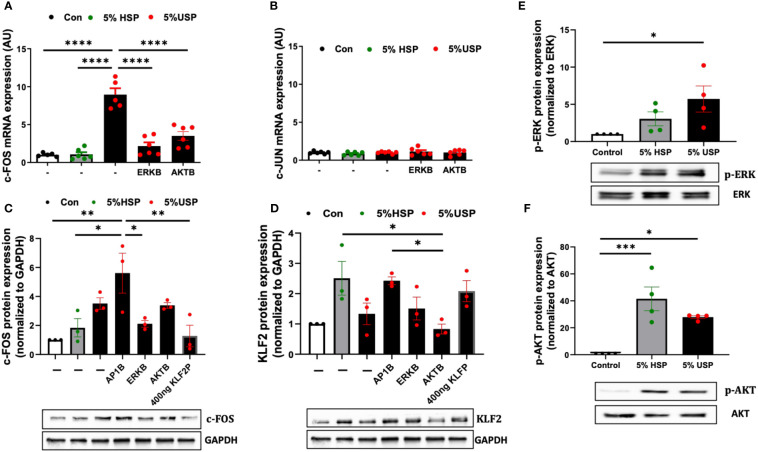
USP-induced KLF2 inhibition engages ERK and AKT signaling *via* c-FOS. **(A, B)** Engagement of c-FOS vs. c-JUN: qRT-PCR quantification of mRNA expression levels (AU, n=6) of the key transcription factors c-FOS and c-JUN upon a 6-hour stimulation with either 5% USP or 5% HSP, with or without 1-hour preincubation with blockers of AKT or ERK signaling pathways: protein kinase B (AKTB: MK-2206, 100 nM), or map-kinase/extracellular signal-regulated kinase (MAPK/ERKB: PD-184352, 100 nM), respectively, and **(C, D)** c-FOS and KLF2: Western blot quantification of protein expression levels for KLF2 and c-FOS (AU, relative to GAPDH, n=3 each) after a 12-hour stimulation with either 5% USP or 5% HSP, following a 1-hour preincubation of HMECs with blocking agents against either activator protein 1 (AP1B: SR-11302, 1000 nM), (AKTB: MK-2206, 100 nM), or (MAPK/ERKB: PD-184352, 100 nM), respectively. **(E, F)** Engagement of p-AKT and p-ERK: Western blot quantification for detection of phosphorylated p-AKT and p-ERK, normalized relative to their non-phosphorylated forms (Ratio of pERK/ERK and pAKT/AKT, n=4), upon a 12-hour stimulation with either 5% USP or 5% HSP. Statistical comparison with ANOVA, Mean ± SEM, with *P<0.05, **P<0.01, ***P<0.001, and ****P<0.0001, ns, not significant.

First, we studied the mRNA expression of c-FOS and c-JUN in response to USP in combination with pharmacological blockers of AKT and MAPK/ERK (**Figures 4A, B**). We found that c-FOS mRNA was strongly upregulated in response to stimulation with 5% USP (P<0.001, **Figure 4A**), while c-JUN mRNA was not affected (not significant, **Figure 4B**). Furthermore, the upregulation of c-FOS mRNA could be inhibited by blocking both AKT and MAPK/ERK signaling pathways (P<0.001, **Figure 4A**). This indicates that AP-1/c-FOS, but not AP-1/c-JUN, is the key transcription factor complex involved in uremic induction.

Next, we studied the protein levels of c-FOS and KLF2 in response to USP-stimulation in combination with using specific AP-1, AKT, and MAPK/ERK pathway blocking agents (**Figures 4C, D**). Interestingly, the preincubation of HMECs with AP-1-blocker resulted in a 2-fold amplification of USP-induced c-FOS protein expression compared to HSP or baseline control (P<0.05 and P<0.01, **Figure 4C**), and the AP-1-blocker could also completely reverse the USP-induced downmodulation of KLF2 protein expression (**Figure 4D**).

Similar to preventing upregulation of c-FOS mRNA expression (P<0.001, **Figure 4A**), the blockade of MAPK/ERK-signaling was more effective in preventing upregulation of c-FOS protein upon USP-induction (P<0.05, **Figure 4C**), while the AKT-blocker was less effective on c-FOS protein than mRNA level, thus indicating that USP-induced c-FOS signaling is more dependent on ERK than AKT signaling. The upregulation of c-FOS protein by USP could be lowered to HSP baseline by transfection of 400 ng KLF2 plasmid (P<0.01, **Figure 4C**), which could also fully restore KLF2 protein expression (**Figure 4D**).

Interestingly, stimulation with 5% USP induced a 5-fold increase in phosphorylation of ERK1/2 compared to baseline (P<0.05, **Figure 4E**), which was approximately 2-fold higher than for stimulation with 5% HSP. In contrast, stimulation with 5% USP induced a 2-fold lower expression of phosphorylated AKT compared to stimulation with 5% HSP (P<0.05 and P<0.001, **Figure 4F**). Thus, USP and HSP are acting directly opposed on ERK and AKT phosphorylation in HMECs, which may explain the results obtained in **Figures 4A–D**.

In summary, the diverse components contained in USP compared to HSP (e.g. higher levels of uremic mediators and toxins with on overall more dysbalanced profile vs. healthy blood environment containing less inflammatory or much less uremic mediators) may result in triggering of multiple signaling cascades (e.g. HMEC response to uremic vs. healthy serum). Thereby, USP and HSP trigger differential AP-1/c-FOS signaling through engaging the KLF2 promoter region, and consequently AKT and MAPK/ERK1/2 canonic signaling pathways, in controlling the expression and regulation of KLF2 expression.

### 3.4 Uremic serum induces a proinflammatory response in HMECs

Endothelial microinflammation and its beneficial modulation during RRT is critical to the pathophysiology and management of uremia in CKD and ESRD (7). Thus, we studied in representative fashion, the expression changes of a panel of typical inflammatory mediators (e.g. pro/anti-inflammatory cytokines and chemokines) in HMECs in response to stimulation with USP vs HSP, by using the well-defined PCR array technology (33–35).

**Figure 5:**
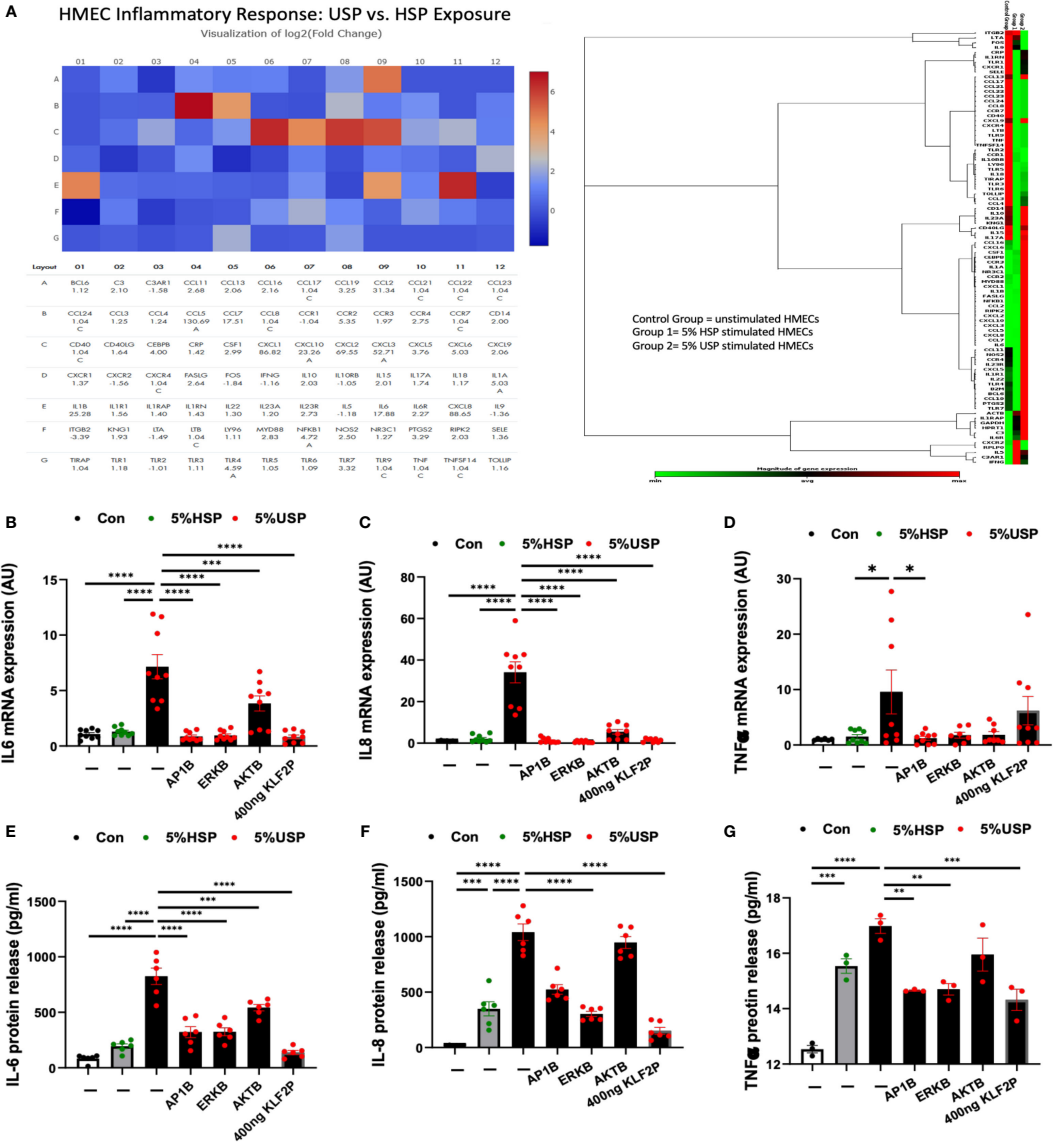
KLF2 modulates USP-induced inflammatory response and cytokine release. **(A)** QPCR-Array Readout: differential proinflammatory response of HMECs upon a 6-hour exposure to either 5% USP or 5% HSP, depicting on the left a Heatmap visualization of log2-fold change for n=84 selected genes typically involved in the inflammatory response and to the right the corresponding gene expression cluster analysis to verify relevant inflammatory gene families associated with USP vs HSP stimulation in HMECs; and **(B–G)** Analysis of Key Inflammatory Markers: **(B, D, F)** qRT-PCR analysis of mRNA expression and **(C, E, G)** ELISA quantification of cytokine release for three selected key cytokines (IL-6, IL-8, and TNF-α, respectively) measured after a 6-hour or 24-hour stimulation, respectively, with either 5% USP or 5% HSP, with or without 1-hour preincubation of HMECs with AP1B, ERKB and AKTB blockers. Statistical comparison with ANOVA, Mean ± SEM, with *P<0.05, **P<0.01, ***P<0.001, and ****P<0.0001. ns, not significant.

Out of a panel of 84 target genes (**Figure 5A**, left panel) a number of proinflammatory activation markers were upregulated in the USP-treated compared to the HSP-treated group (**Figure 5A**, right panel, cluster analysis: group 2 upregulated transcripts shown in red vs. group 1 unchanged or downmodulated transcripts shown in green, vs. non-treated baseline control group), including targets such as IL-6, CCL2, CCL5, CCL7, CCR2, CXCL1,CXCL8, NFKB1, TLR4 et, while ITGB2 was downregulated compared to the HSP group.

To quantify the changes in specific targets, we measured representative cytokines on mRNA and protein level (e.g. IL6, IL8 and TNF-a, **Figures 5B–G**) in the presence or absence of the blocking agents or KLF2 plasmid. Indeed, we could confirm that IL-6, IL8, and TNF-a are strongly upregulated in HMECs upon incubation with 5% USP compared to 5% HSP or untreated baseline, on mRNA level (P<0.05 to P0.0001, **Figures 5B, D, F**) and also on the protein level (P<0.01 to P<0.0001, **Figures 5C, E, G**).

Incubation with blocking agents directed against AP-1, AKT, or MAPK/ERK1/2 was effective in downmodulating the proinflammatory cytokine mRNA production (P<0.05 to P<0.0001, **Figures 5B, D, F**), and in particular AP-1- and ERK-blockers were effective in also downmodulating the respective protein production (P<0.01 and P<0.0001, **Figures 5C, E, G**). The AKT-blocker was effective in blocking IL-6 protein release (P<0.001, **Figure 5C**), but less effective in blocking IL-8 and TNF-a production (both not significant).

The introduction of the KLF2 overexpression plasmid strongly reduced HMECs’ mRNA production of IL-6 and IL-8 in presence of 5% USP (P<0.0001, **Figures 5B, D**), while TNF-a mRNA expression was unchanged (**Figure 5F**). The protein production of all three mediators, IL-6, IL-8, and TNF-a was strongly downmodulated upon introduction of KLF2 plasmid (**Figures 5C, E, G**).

### 3.5 Compared to HF dialyzers, expanded hemodialysis therapy with novel improved MCO dialyzers normalizes KLF2 transcription in endothelial cells

Our experimental results demonstrate, that USP exposure promotes the inhibition of KLF2 transcription in HMECs by engaging the AP-1/c-FOS transcription factor complex, with activation of ERK and AKT signaling, which is followed by the induction of typical proinflammatory markers such as IL-6. In turn, the blockade of AP1/ERK/AKT-signaling or supplementation of KLF2 overexpression plasmid could reverse the USP-induced activation of inflammatory markers. We next wondered how this would translate in the clinical setting.

**Figure 6:**
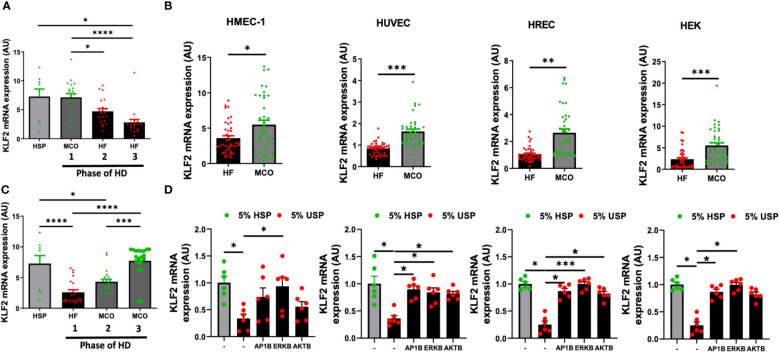
Expanded hemodialysis (HDx) therapy with novel improved MCO dialyzers normalizes KLF2 transcription in HMEC, HUVEC, HREC, and HEK cells, by modulating AP-1/c-FOS and AKT/ERK-dependent intracellular signaling. The effect of uremic serum fractions collected from patients undergoing hemodialysis with two different dialyzers/dialysis regimens in the PERCI-II clinical trial on KLF2 expression in different endothelial cell types. The serum fractions were collected from patients first dialyzed with high flux (HF) dialysis during the run-in phase of the trial and then dialyzed either with the conventional HF or the novel improved medium cut-off (MCO) dialyzer for comparison of the two different dialysis regimens in a cross-over clinical study design (n=40 patients included in analysis, comparison of 20 patients in each phase, the inclusion target was 50 patients) (7, 17). **(A, B)** KLF2 mRNA transcript levels at different trial stages were measured after stimulation of HMECs with healthy serum pool HSP reference or individual uremic serum samples from different stages of the PERCI-II trial (n=20 per group), as we previously described in PERCI-II study (A: MCO/HF/HF vs. B: HF/MCO/MCO) (7, 17), **(C, D)** To study if this can be generalized to other cell types, we also compared the effect of HF and MCO on HMECs, HUVECs, HRECs, and HEKs in presence or absence of different inhibitors (AP1B, ERKB, AKTB). KLF2 mRNA transcript levels were measured after a 6-hour incubation of HMECs with 5% uremic serum (n=40 patients) collected at the end of the first HF or MCO phase (each cycle lasted 4 weeks), using ANOVA, Mean ± SEM, with *P<0.05, **P<0.01, ***P<0.001, and****P<0.0001.

We have previously already demonstrated in the PERCI-II clinical study that novel improved medium cut-off (MCO) dialyzers reduce uremic serum (USP)-induced systemic micro-inflammation and endothelial dysfunction (7, 17). Intriguingly, we can here report for the first time that this also translates into beneficial modulation of KLF2 expression (**Figure 6**).

We first of all found that exposure of HMECs to uremic serum from patients treated either with conventional high-flux (HF) or novel improved MCO dialyzers resulted in normalization of KLF2 mRNA expression with MCO dialyzers (P<0.5 to P<0.001, **Figures 6A, B**).

When analyzing the individual trial phases, we first of all found that HMECs incubated with uremic serum from patients in the MCO/HF/HF-regimen, starting with MCO dialyzer, had similar KLF2 expression upon incubation MCO serum (end of the first 4-weeks cycle) than upon incubation with HSP, which indicates a beneficial effect of the MCO dialyzer (**Figure 6A**). In turn, the incubation of HMECs with uremic serum from the following two conventional HF cycles (4 and 8 weeks, respectively, in total 12 weeks long) lead to a strong downmodulation of KLF2 expression in the HMECs (P<0.05 to P<0.0001, **Figure 6A**).

Vice versa, HMECs incubated with uremic serum from patients in the cross-over HF/MCO/MCO-regimen, starting with HF dialyzer, had a strong downmodulation of KLF2 mRNA expression upon incubation of HMECs with the HF uremic serum collected at the end of the first 4-weeks cycle (P<0.0001, **Figure 6B**). However, incubation of HMECs with uremic serum from the following two MCO cycles (again 4 and 8 weeks, respectively) was effective in normalizing KLF2 expression to the prior baseline levels observed with HSP (P<0.001 and P<0.0001, **Figure 6B**).

We next wondered if similar protective effects of MCO-dialysis could be observed with other commonly used endothelial and kidney model related cell types, such as HUVECs, HRECs, and HEK cells (**Figures 6C, D**). Indeed, we found that in pair-wise analysis improved KLF2 expression could be observed in HMECs, HUVECs, HRECs, and HEKs (P<0.05, P<0.001, P<0.001, and P<0.01, respectively, **Figure 6C**), comparable to that of HMECs.

In addition, we found that similarly to the signaling pathways employed in HMECs, any detrimental endothelial down-modulation of KLF2 upon exposure to conventional uremic serum from HF dialysis could be mostly reversed by employing selective AP-1-, ERK-, and AKT-blockade in HUVECs, HRECs, and HEKs, respectively (P<0.05 to 0.001, **Figure 6D**). The ERK-blockade was slightly more effective than the AKT-blockade, thus demonstrating a more dominant signaling through the AP-1/ERK pathway (**Figure 6D**). Intriguingly, the dominance of ERK signaling was strongest in the HMECs, while this was less evident in the other three cell lines tested, although in general similar trends considering the dominance of AP-1/ERK signaling could be observed (**Figure 6D**).

In conclusion, this indicates that MCO dialyzers may offer a better treatment outcome compared to the HF dialyzer, through normalizing KLF2 transcription, which is important for mediating anti-inflammatory and angiogenic effects.

## 4 Discussion

High cardiovascular morbidity and mortality, caused in part by chronic inflammation associated with endothelial dysfunction, remains a major challenge in the management of CKD and ESRD patients with underlying uremia and systemic microinflammation (8–12). Endothelial cells (ECs) - as a direct barrier to the uremic substances in the blood - are essential for maintaining vascular homeostasis (49). There is an urgent unmet medical need to reverse endothelial dysfunction and reduce chronic inflammation in the clinical setting of renal replacement therapies (RRTs) (7–13). Thus, we here conducted a detailed study to decipher the major role of KLF2, a key vascular mediator and regulator of inflammatory process (20).

Krüppel-like factor 2 (KLF2), a key transcription factor involved in anti-inflammatory processes in the vasculature, has been proposed to be a critical target in the pathobiology of both CKD and ESRD (39, 50). Intriguingly, the effect of uremic serum (in particular uremic serum from well controlled clinical trials, such as PERCI-I/II) on the regulation and signaling of KLF2 has not been explicitly studied to date. Therefore, we focused in the present study on investigating uremic serum induced transcriptional regulation of KLF2 expression and the engagement of concomitant signaling pathways in microvascular endothelial cells (HMECs), since these cells are typically also a major target of uremic microinflammation (7).

First of all, we found that exposure of HMECs to uremic serum inhibits KLF2 mRNA transcription *via* engaging activating protein-1 (AP-1)/c-FOS signaling, thus resulting in the activation of several canonical signaling pathways, such as MAPK/ERK and AKT signaling, which in turn leads to a strong release of proinflammatory mediators by HMECs, including interleukin-6 (IL-6) and IL-8 and a panel of chemokines, among others. This is in line with our prior results on the response of ECs to uremic microinflammation in the context of endothelial maladaptation (7). Linking the modulation of KLF2, a key vascular transcription factor, to this study provides further insight into the mechanisms underlying the beneficial effect of expanded hemodialysis with improved medium cut-off (MCO) dialyzers (8–12).

Indeed, KLF2 expression is essential for renal vascular integrity and function (20, 39), and upregulation of KLF2 restores glomerular endothelial damage in mice undergoing unilateral nephrectomy (51). In turn, reduced expression of KLF2 potentiates podocyte injury in diabetic nephropathy and increases renal proteinuria (27, 52). Loss of KLF2 expression is also associated with leading to excessive accumulation of uremic solutes in patients with ESRD (53, 54). In support of these results, our current study found a substantial dose- and time-dependent repression of KLF2 transcriptional activity and protein expression in USP-treated HMECs. Thus, chronic accumulation of uremic toxins and inflammatory mediators in USP can lead to a loss of protective anti-inflammatory KLF2 expression in ECs exposed to uremia.

The regulation of KLF2 is complex and may be orchestrated by a variety of genes (39). Thus, the observed downmodulation of KLF2 in response to USP may be coregulated by a multiplicity of genes and signaling pathways. Indeed, biologically, it is not uncommon for genes to share or co-ordinate with similar regulatory binding elements to counter-influence transcriptional regulation. Interestingly, a comparative *de-novo* analysis of podocyte slit diaphragm molecules revealed that many genes share KLF promoter binding sites (55).

Similarly, downregulation of KLF2 transcription is known to up-regulate several genes and transcription factors such as Binding Endothelial Cell Precursor-Derived Regulator (BMPER) and Suppressor of Mothers against Decapentaplegic (SMAD1) (56, 57), thus also suppressing vascular calcification (57). However, the KLF2 promoter sequence to which regulatory factors bind is still not studied in great detail. Interestingly, an anticalcifying effect on vascular smooth muscle cells (VSMCs) was also reported for the use of uremic serum obtained from patients dialyzed with high- and medium-cut-off HCO/MCO dialyzers (58, 59).

Indeed, early reports suggested that KLF2 promoter activity is influenced by histone methylation or acetylated histones H3 and H4 associated with bare stress or other signals (60, 61). In addition, nuclear factors, including heterogeneous nuclear ribonucleoprotein (hnRNP)-U, hnRNP-D, and P300/CBP-associated factor (PCAF), can also bind to the human KLF2 promoter region (in position -138 and -111 bp) and regulate its transcription (62), while sequestration of the transcriptional coactivator p300/CBP by KLF2 suppresses inflammation through preventing NF-kB/p300 interaction and subsequent activation of the vascular cell adhesion molecule-1 (VCAM-1) (20, 41).

KLF2-p300 interaction also permits KLF2 binding to the endothelial nitric oxide synthase (eNOS) promoter to induce the transcription of this vasculoprotective enzyme (41). These early studies demonstrated KLF2’s ability to influence transcription through both, direct DNA-binding and/or indirect cofactor sequestration mechanisms (20). Furthermore, the KLF2 promoter region from -157 to -95 bp is also essential for the induction of fluid shear stress in the endothelium (63). In addition, p53 can also target the KLF2 promoter and repress its transcription in microglial cells (64).

In our study, USP-stimulated HMECs were found to downregulate KLF2 expression through the engagement of the KLF2 promoter sequence region -509 to -109 as a crucial regulatory region. Interestingly, we identified an AP-1 binding site in this region and studied, whether AP-1 is involved in the regulation of KLF2 transcription. Previously, KLF2 was shown to be involved in endothelial inflammation by negatively regulating AP-1 (28), but the specific promoter binding region was unknown. Indeed, targeted deletion analysis in our study indicates that AP-1 and the constitutive element c-FOS rather than c-JUN bind to the KLF2 promoter sequence -464 to -441 to regulate the inhibition of KLF2 in uremia.

Transcription of KLF2 is regulated by proliferative and inflammatory pathways (21, 41). Independent studies have demonstrated the regulation of KLF2 by mitogenic markers, e.g. MAPK/ERK-signaling or through its specific inhibitor PD184352 in mast cells (65). Interestingly, glucose-loading of human umbilical vein endothelial cells (HUVECs) also represses KLF2 transcription *via* attenuated ERK 1/2 activation (66). Shimizu et al. reported that the uremic toxin indoxyl-sulfate can increase monocyte chemoattractant protein-1 (MCP-1) production by activating reactive oxygen species (ROS) and the MAPK/ERK1/2 signaling (67).

MAPK/ERK-signaling is involved in a ricin-induced severe inflammatory response in mouse hemolytic-uremic syndrome in mice (45), thereby suggesting that ERK-signaling may act as a deleterious mediator in the response to uremic toxins. Similarly, enhanced phosphorylation of *p-*AKT has been observed in different stages of both, hemodialysis- and peritoneal-dialysis-treated uremia (68). Interestingly, the uremic toxin indoxyl-sulfate was found to suppress the expression of *p*-AKT, but not ERK in HUVECs (69), while P-cresol, another uremic toxin, was found to activate *p*-AKT and *p*-ERK in VSMCs (70), indicating a certain cell type dependency.

The molecular analysis in our current study indicates, that there exits some crosstalk between USP-stimulation-induced regulation of KLF2 transcription and both ERK and AKT pathway activation. This crosstalk is at least in part mediated *via* AP-1/c-FOS-signaling. Indeed, the suppression of mRNA expression of c-FOS, but not c-JUN, by pharmacological inhibition of ERK-signaling clearly indicated that c-FOS is crucially involved in USP-induced inhibition of KLF2 transcription. Binding of the AP-1/c-FOS complex to the KLF2 promoter region appears to mediate both, the activation of ERK and AKT pathways. Nevertheless, global transcriptomic analysis of KLF2 signaling may be necessary to further uncover additional potential DNA binding elements of the KLF2 promoter sequence.

We found that optimized amounts of KLF2 plasmid transfected into HMECs could attenuate the negative effects of USP-induced proinflammatory cytokine release, e.g. including IL-6, IL-8, and TNF-α. Remarkably, exogenous KLF2 could also inhibit c-FOS expression, indicating a concurrent effect on other regulatory signaling mechanisms, such as ERK and AKT activation. Thus, targeting the modulation of endogenous KLF2 expression (or its activity) could motivate novel therapies to treat the vascular microinflammation induced by uremia. Indeed, several pharmacological approaches, e.g. the use of statins, suberanilohydroxamic acid, tannic acid, and resveratrol, have all been shown to exert atheroprotective and anti-inflammatory effects on the endothelium through induction/targeting of KLF2 (19, 71–75).

The effect of USP on the inhibition of KLF2 mRNA transcription suggested, that dialysis membranes with improved molecular cutoff could offer a better therapeutic outcome. To answer this question, we retrospectively studied USP of patients treated with either high-flux (HF) or medium-cut-off (MCO) dialyzers (7, 41), observing significant improvements in the expression of KLF2 in the MCO group, which is in line with improvements in other inflammatory pathways and reduced endothelial maladaptation (7). This suggests an improved effectiveness of MCO to not only remove small toxic molecules (<0.5kD), but also middle-sized molecules and pro-inflammatory mediators, such as TNF-α, interleukins, β-2-microglobulin, and others (76), compared to the conventional HF hemodialyzers.

In agreement with our prior study (7), MCO may offer a better vasculoprotective role through improved maintenance/upregulation of KLF2 transcription in uremic patients. Indeed, prolonged HD with MCO (for up to 21 weeks) restored KLF2 expression in USP-stimulated HMECs to that found in HMECs stimulated with HSP, which may indicate a beneficial effect for the respective MCO-treated patients. Conversely, extended application of HF gradually reduced KLF2 to pre-treatment levels. Thus, our study demonstrates that uremic solutes down-regulates KLF2 transcription, leading to upregulation of proinflammatory mediators such as IL-6 *via* triggering AP-1/c-FOS, ERK and AKT signaling. However, maintaining/restoring KLF2 expression levels by use of beneficial MCO dialyzers (or exogenous KLF2 induction) could be a novel treatment approach to restore renal integrity and function in CKD/ESRD.

## 5 Conclusions and study limitations

Here, we identified that uremia downmodulates vasculoprotective KLF2 expression in common endothelial/kidney model cell lines, such as HMECs, HUVECs, HRECs, and HEKs. We then examined in detail the regulatory signaling mechanisms underlying modulation of KLF2 expression in response to uremic serum (USP). First, we identified the signaling events triggered upon USP-stimulation, including AP-1/c-FOS, MAPK/ERK1/2 and AKT signaling, as respective upstream molecules to regulate the expression of KLF2 in HMECs, and found that this was also applicable to other typical cell lines, such as HUVECs, HRECs, and HEKs.

Next, we demonstrated that any adverse effects of USP-exposure can be effectively attenuated by modulating endogenous KLF2 expression, which was achieved by specific inhibitors or transfection with an exogenous KLF2 plasmid. Further, we compared the inflammatory response to uremic and healthy serum, identifying a panel of mediators involved in uremia-induced chronic vascular inflammation.

Importantly, clinical verification of our results in a well-controlled clinical trial cohort, dialyzed with novel improved medium-cutoff (MCO)-dialysis membranes, was found to better maintain, or even restore KLF2 expression (cross-over design). Indeed, prolonged MCO dialysis eliminated any prior downmodulation of endothelial KLF2 expression observed with conventional HF dialysis membranes or uremic serum before HD treatment.

Thus, novel clinical trials with focus on comparing the therapeutic effects of MCO and HF membranes, but also well-designed pharmacological studies to targeting the respective signaling pathways, may provide new insights for future research and future improvements for patients depending on RRTs, to further reduce the detrimental long-term effects of uremia, chronic vascular inflammation and concomitant organ damage.

## Data availability statement

The raw data supporting the conclusions of this article will be made available by the authors, without undue reservation.

## Ethics statement

The studies involving humans were approved by Ethics Committees of the Martin-Luther-University Halle-Wittenberg and the Charité Berlin. The studies were conducted in accordance with the local legislation and institutional requirements. The participants provided their written informed consent to participate in this study.

## Author contributions

Experiments and investigation: HZ, DW, MG, CL, PW, AP. Writing, review, and editing: HZ, DW, MA, JK-M, JW, GM, RC. Conception and Supervision: JW, DD, KB, RS, DZ, GM, RC. All authors contributed to the article and approved the submitted version.

## Supplementary material

Supplementary Figure 1 Analysis of the KLF2 promoter region and AP-1 binding site.
