# Supplementary figures and images for: Expanded Hemodialysis ameliorates uremia-induced impairment of vasculoprotective KLF2 and concomitant proinflammatory priming of endothelial cells through an ERK/AP1/cFOS-dependent mechanism
Source: Front Immunol. 2023 Sep 19;14:1209464. doi: 10.3389/fimmu.2023.1209464 (PMC10546407; doi:10.3389/fimmu.2023.1209464)

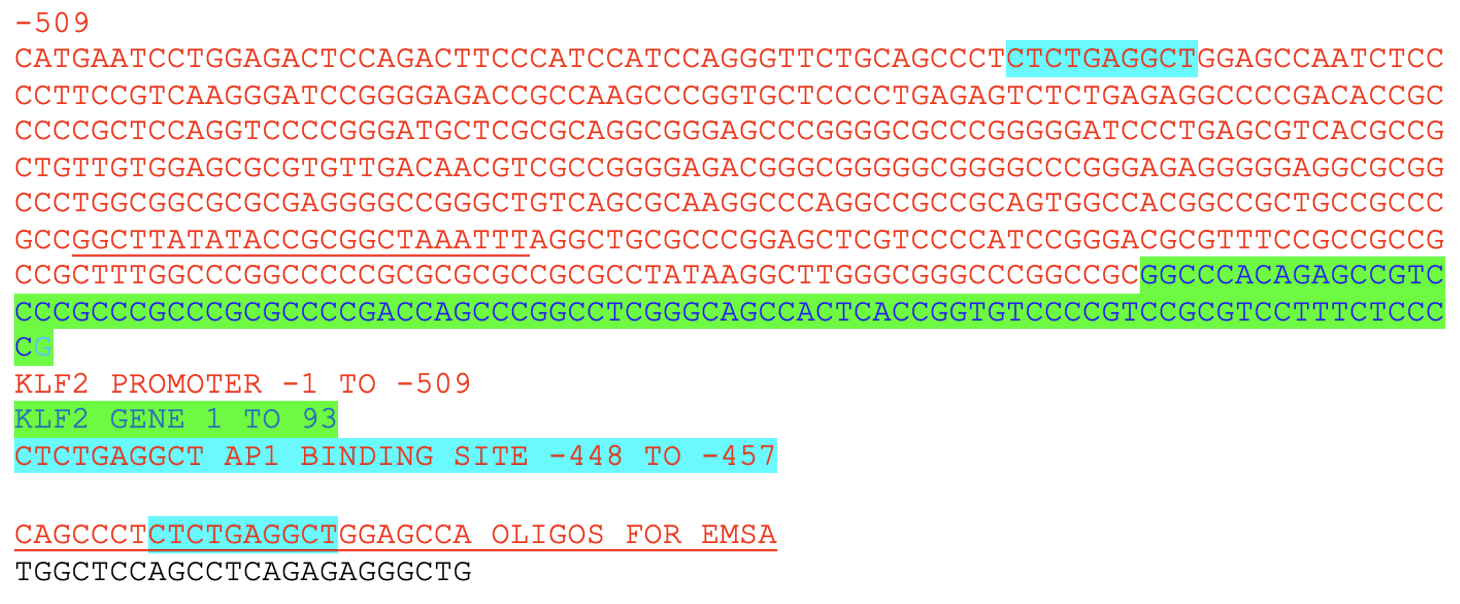

Supplement: Supplementary Figure 1 — Analysis of the KLF2 promoter region and AP-1 binding site. [file Image_1.tif]
